# Irradiance footprint of phototherapy devices: a comparative study

**DOI:** 10.1038/s41390-021-01795-x

**Published:** 2021-11-02

**Authors:** Alida J. Dam-Vervloet, Nienke Bosschaart, Henrica L. M. van Straaten, Lieke Poot, Christian V. Hulzebos

**Affiliations:** 1 Medical Physics Department, Isala Hospital, Zwolle, The Netherlands; 2 Biomedical Photonic Imaging group, Technical Medical Centre, University of Twente, Enschede, The Netherlands; 3 Neonatology Department, Isala Hospital, Zwolle, The Netherlands; 4 Neonatology Department, UMCG, Groningen, The Netherlands

## Abstract

**Background:**

Phototherapy (PT) is the standard treatment of neonatal unconjugated hyperbilirubinemia. The irradiance footprint, i.e., the illuminated area by the PT device with sufficient spectral irradiance, is essential for PT to be effective. Irradiance footprint measurements are not performed in current clinical practice. We describe a user-friendly method to systematically evaluate the high spectral irradiance (HSI) footprint (illuminated area with spectral irradiance of ≥30 μW cm^−2^ nm^−1^) of PT devices in clinical practice.

**Materials and methods:**

Six commercially available LED-based overhead PT devices were evaluated in overhead configuration with an incubator. Spectral irradiance (µW cm^−2^ nm^−1^) and HSI footprint were measured with a radiospectrometer (BiliBlanket Meter II).

**Results:**

The average measured spectral irradiance ranged between 27 and 52 μW cm^−2^ nm^−1^ and HSI footprint ranged between 67 and 1465 cm^2^, respectively. Three, two, and one PT devices out of six covered the average BSA of an infant born at 22, 26–32, and 40 weeks of gestation, respectively.

**Conclusion:**

Spectral irradiance of LED-based overhead PT devices is often lower than manufacturer’s specifications, and HSI footprints not always cover the average BSA of a newborn infant. The proposed measurement method will contribute to awareness of the importance of irradiance level as well as footprint measurements in the management of neonatal jaundice.

**Impact:**

- While a sufficient spectral irradiance footprint is essential for PT to be effective, some PT devices have spectral irradiance footprints that are too small to cover the entire body surface area (BSA) of a newborn infant.
- This study introduces a user-friendly, accessible method to systematically evaluate the spectral irradiance level and footprint of PT devices.
- This study supports awareness on the role of the spectral irradiance footprint in the efficacy of PT devices. Irradiance footprint can be easily measured during phototherapy with the proposed method.

## Introduction

Neonatal jaundice, resulting from unconjugated hyperbilirubinemia, is a common phenomenon, that may either be relatively benign or progress to potential harmful conditions, i.e., acute bilirubin encephalopathy and kernicterus spectrum disorders (KSDs).^1^ Phototherapy (PT) is considered a safe and effective treatment for neonates of more than 35 weeks gestation with severe hyperbilirubinemia. PT reduces total serum bilirubin (TSB) levels and the need for exchange transfusions (ETs).^2^ Appropriate spectral irradiance is essential for PT to be effective.^3^ The American Academy of Pediatrics recommends at least 30 μW cm^−2^ nm^−1^ for intensive PT that should be delivered to as much of the infant’s surface area as possible.^4^ The efficacy of PT is determined by user factors, technical factors of the PT device, and patient characteristics. User factors relate to: (1) the duration of the PT treatment; (2) distance between the PT device and the infant; (3) effective irradiated body surface area (BSA), which is influenced by the presence of diapers, electrode patches, etcetera.^2,5^ Technical factors of the efficacy of PT relate to: (1) spectral emission range and peak wavelength (nm); (2) spectral irradiance level; (3) irradiance footprint; and (4) uniformity of the spectral irradiance.^2,6–10^ These technical factors are affected by device type, spectral irradiance settings, age, intensity of use and status of maintenance. Patient characteristics include severity of hyperbilirubinemia, BSA, pigmentation, thickness and blood perfusion of the skin.^11–13^ Several studies show high variability in spectral irradiance between PT devices. In these studies, user factors and technical factors were varied simultaneously.^14,15^ Distinction between these factors may enable customized use of a PT device and facilitate comparison between PT devices.

The American Academy of Pediatrics (AAP) recommends to average spectral irradiance measurements at multiple sites, because of a decay in spectral irradiance towards the periphery of the illuminated area.^4^ The number of spectral irradiance measurements and measurement locations are not mentioned. Manufacturers of PT devices use a variety of methods for irradiance measurements. This hinders an objective comparison between spectral irradiance based on technical specifications.^16–21^

Various authors stress the importance of spectral irradiance measurements during PT that enables to adjust spectral irradiance to desired intensity levels.^4,6,9,10,14,15,22^ Increasing the spectral irradiance of a PT device produces a faster decline of TSB levels.^23–27^ Decades ago, Tan suggested a saturation point of 30 μW cm^−2^ nm^−1^ beyond which an increase in the spectral irradiance would not increase efficacy of PT.^25^ More recently, Vandborg et al.^24^ found a linear relation between TSB decline and spectral irradiance between 20 up to 55 μW cm^−2^ nm^−1^. Moreover, the greater the exposed BSA, or the related spectral irradiance footprint of a PT device, the larger the rate of decline of TSB levels.^8,25,27^ Although the spectral irradiance footprint is essential for PT to be effective, how to measure this footprint is not included in hyperbilirubinemia guidelines and is not performed in current clinical practice. Vreman et al.^23^ and Hart and Cameron^7^ used a meticulous method to relate spectral irradiance footprint of a PT device to BSA of a (pre)term newborn infant, with, respectively, 228 and 648 measurement locations. A faster method to perform spectral irradiance of PT devices is suggested by Reda,^28^ which developed an PT spectral irradiance measuring device which automatically performs 15 spectral irradiance measurements.^28^ No previous research investigated the irradiance footprint related to the minimal spectral irradiance of 30 μW cm^−2^ nm^−1^, hereinafter referred to as “high spectral irradiance (HSI) footprint” of a PT device.

To promote irradiance measurements of PT devices in clinical practice, this study aims to introduce a user-friendly and accessible method to systematically evaluate the spectral irradiance level and HSI footprint of six overhead LED-based PT devices.

## Methods and materials

### PT devices

For this study, we evaluated six LED-based PT devices that are commercially available: BlueSpot (GE Healthcare, Wauwatosa), Infantulus (Arseus Hospital, Bornem, Belgium), Bililux (Dräger, Lübeck, Germany), Lullaby (GE Healthcare, Wauwatosa), NeoBlue Compact (Natus Medical, Middleton), and the BiliblueLight (Löwenstein Medical, Bad Ems, Germany). Four PT devices (Infantulus, Bililux, Lullaby, NeoBlue Compact) provided different spectral irradiance settings with adjustable light intensities, which we also evaluated in this study. Table 1 shows the spectral emission range and spectral irradiance according to the manufacturers’ specification.^16–21^ All PT devices were tested at the same location (Isala Hospitals, Zwolle, The Netherlands).

**Table 1:** Included PT devices, PT device spectral irradiance setting (PT-device setting), spectral irradiance according to specifications of the manufacturer, average measured spectral irradiance, measured spectral irradiance uniformity, and measured intra-device reproducibility.

Type PT device	PT-device setting	Spectral emission range according specs (nm)	Spectral irradiance according specs (µW cm^−2^ nm^−1^)	Average measured spectral irradiance (µW cm^−2^ nm^−1^)	Measured spectral irradiance uniformity (µW cm^−2^ nm^−1^) min max SD			Measured intra-device reproducibility (µW cm^−2^ nm^−1^)
					Min	Max	SD	
1. BlueSpot (GE) (16)	**n.a**.	**400–520**	**40**	**42**	**39**	**44**	**1.2**	**0.4**
2. Infantulus (Arseus Hospital) (17)	**level 3**	**445-450**	**65**	**52**	**30**	**133**	**41.2**	**0.5**
	level 2		50	33	20	82	24.9	0.4
	level 1		35	24	13	61	18.4	0.5
3. Bililux (Dräger) (18)	**level 100%**	**460–490**	**50**	**39**	**36**	**42**	**2.4**	**0.2**
	level 80%		_	31	29	34	2.0	0.1
	level 60%		_	23	21	25	1.4	0.1
	level 40%		_	16	14	18	1.4	0.0
	level 20%		_	7	7	8	0.6	0.0
4. Lullaby (GE) (20)	**level 2**	**450–465**	**45**	**37**	**33**	**43**	**3.4**	**0.2**
	level 1		22	17	15	20	1.6	0.1
5. NeoBlue Compact (Natus) (19)	**level High**	**450–470**	35	**29**	**26**	**34**	**2.9**	**0.1**
	level Low		15	13	12	15	1.2	0.1
6. BiliblueLight (Löwenstein) (21)	**n.a.**	**440–460**	**45**	**27**	**25**	**31**	**2.1**	**0.1**

*n.a. not applicable.

The highest spectral irradiance settings per PT device are highlighted in bold.

### Spectral irradiance measurements

Spectral irradiance (μW cm^−2^ nm^−1^) measurements were made using a calibrated BiliBlanket Meter II (GE Healthcare, Fairfield). This spectroradiometer was selected because it is one of the most commonly used devices and it is sensitive over a wide spectral range (400–520 nm with peak sensitivity at 450 nm) and dynamic range (0.1–299.9 μW cm^−2^ nm^−1^).^29^ The spectral range and dynamic range of this spectroradiometer overlap with the bilirubin absorption spectrum^23^ and the PT delivery range,^16–21^ respectively. Spectral irradiance is reported as whole integers.

### Experimental setup

**Fig. 1:**
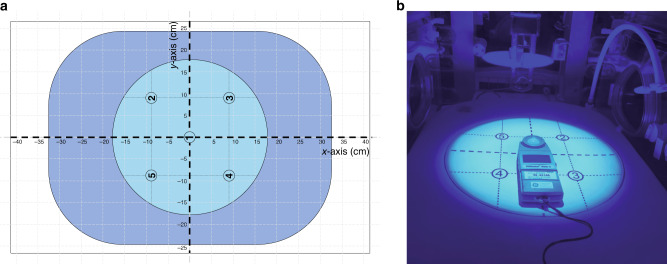
Measurement setup. **a** Schematic top view of the measurement setup was the measurement grid with five irradiation measurement locations (light blue) is centered on the mattress (dark blue) in the incubator. **b** Photograph of the spectroradiometer placed in the incubator at measurement location 1 of the irradiated measurement grid (device 1, Table 1).

All measurements were performed in an incubator (Giraffe, GE, Waukesha). A measurement grid with five measurement locations, around the chest of a mannequin of a term newborn infant, was centered on the mattress in the incubator, in analogy to a proposed pre-use check out procedure (Fig. 1).^16^ PT devices (Table 1) were configured as recommended by the manufacturers: PT-device 1 was placed above the incubator and PT-device 2–6 were placed on top of the incubator. This resulted in PT device to radiospectrometer distances of 38 cm (device 1) and 35.5 cm (device 2–6). The spectral irradiance was measured repeatedly for five times per PT device, five times per measurement location and at five measurement locations to obtain a good estimate of the spread of spectral irradiance.

### Spectral irradiance

We averaged the measured spectral irradiance levels at all five measurement locations to one average spectral irradiance level for each PT device as recommended by the AAP.^4^ Spectral irradiance uniformity was quantified as the standard deviation (SD) of all spectral irradiance measurements per PT device. The spectral uniformity is considered high if it is ≤10% of the average spectral irradiance.

To adequately evaluate inter-device variability, the intra-device reproducibility of the employed methods must be well known. Intra-device reproducibility was quantified as the averaged SD of all spectral irradiance measurements points per PT device. The intra-device reproducibility was considered high if ≤3% of the measured spectral irradiance conform accuracy specification of the BiliBlanket Meter II.^29^

### High spectral irradiance footprint

To define the HSI footprint area where the spectral irradiance was ≥30 μW cm^−2^ nm^−1^, the radiospectrometer was moved along the *x*- and *y*-axis of the measurement grid (Fig. 1a). The locations where the spectral irradiance became less than 30 μW cm^−2^ nm^−1^ were recorded, resulting in four measurement points: *x*_p_, *x*_n_, *y*_p_, and *y*_n_, where the subscripts p and n denote locations on the positive and negative sides of the axes, respectively. The parameters *d*_*x*_ and *d*_*y*_ were defined as the distance along the *x*- and *y*-axis where the irradiance was ≥30 μW cm^−2^ nm^−1^: *d*_*x*_ = *x*_p_ – *x*_n_ and *d*_y_ = *y*_p_ – *y*_n_. The HSI footprint was calculated by combining the measured values of *d*_x_ and *d*_y_ with the geometry of the irradiance footprint of the PT devices: HSI footprint_round/oval_ = *π*(1/2*d*_*x*_)(1/2*d*_*y*_) for round and oval geometries (Bluespot, Infantulus) and HSI footprint_rectangular_ = *d*_*x*_*d*_*y*_ for rectangular geometries.

The HSI footprint of each PT device was related to the average BSA of an infant born at 22, 26, 32, and 40 weeks of gestation with an average body length of 28, 35, 42, and 50 cm, respectively.^30–32^

## Results

### Spectral irradiance

Table 1 shows the average measured spectral irradiance levels of all individual PT devices. We observed a large variation in the measured spectral irradiance between PT devices. The measured spectral irradiance ranged between 27 μW cm^−2^ nm^−1^ (Bilibluelight) and 52 μW cm^−2^ nm^−1^ (Infantulus). So, the maximal difference in the measured spectral irradiance amounted up to 25 μW cm^−2^ nm^−1^. Both NeoBlue Compact (29 μWcm^−2^ nm^−1^) and Bilibluelight (27 μW cm^−2^ nm^−1^) showed a spectral irradiance of slightly less than the recommended 30 μW cm^−2^ nm^−1^.

Spectral irradiance uniformity was high (SD < 3.5 μW cm^−2^ nm^−1^) for five of the six evaluated PT devices. Only the uniformity of the Infantulus was low (for all different spectral irradiance settings). This low uniformity was caused by a high centered spectral irradiance peak (133 μW cm^−2^ nm^−1^ at location 1) and a relatively rapid decay in spectral irradiance towards the periphery (32 μW cm^−2^ nm^−1^ at locations 2–5), for the highest spectral irradiance setting.

The measured intra-device reproducibility ranged between 0.0 and 0.5 μW cm^−2^ nm^−1^ for all devices. The maximum intra-device reproducibility was observed for the Infantulus.

### High spectral irradiance footprint

**Fig. 2:**
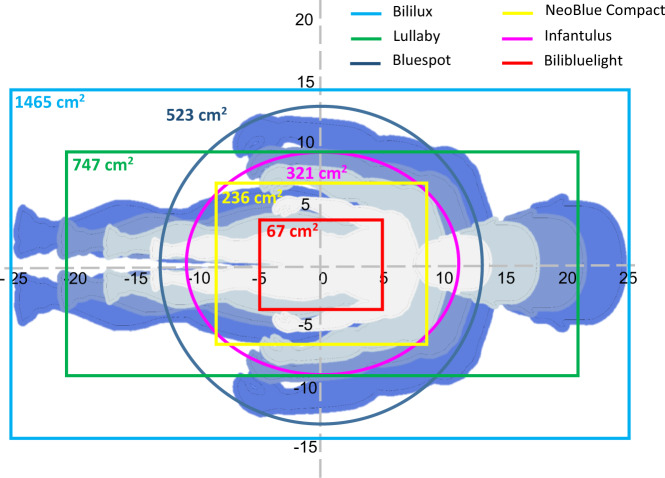
Schematic overview of all PT-irradiance footprints with measured spectral irradiance >30 μW cm^−2^ nm^−1^. High spectral irradiance footprints of the BlueSpot, Infantulus, Bililux, Lullaby, NeoBlue Compact, and Bilibluelight are indicated by dark blue, purple, blue, green, yellow, and red, respectively. The light gray, gray, light blue, and blue silhouettes represent the body surface area of an infant born an average body length of 22, 26, 32, and 40 weeks of gestation, respectively.^30–32^ Silhouettes were adapted and used with permission.^14^

Figure 2 shows the HSI footprints of all PT devices. Only the HSI footprint of the Bililux covered the average BSA of infants born at 40 weeks of gestation. The Lullaby covered the BSA of an infant born at 26 and 32 weeks and the BlueSpot of an infant born at 22 weeks of gestation. Infantulus and NeoBlue Compact cover the largest part of the average BSA of an infant born at 22 and 26 weeks of gestation.

For the highest spectral irradiance settings per manufacturer, the maximal difference in the HSI footprint amounted up to 1398 cm^2^ between Bilibluelight (67 cm^2^) and Bililux (1465 cm^2^).

## Discussion

The main purpose of this study was to evaluate and compare the spectral irradiance levels and HSI footprint of PT devices with a user-friendly method. Hereto, we measured both parameters of six commonly used PT devices of different brands with a radiospectrometer using a measurement grid that consisted of five (spectral irradiance) and four (HSI footprint) measurement locations, respectively. We observed a large variation in the measured spectral irradiance and HSI footprint between PT devices. In this study, we demonstrated that the proposed method can be easily performed to compare technical factors of PT devices.

The maximum encountered spectral irradiance difference between two different PT devices was 25 μW cm^−2^ nm^−1^. Also a large variation between measured spectral irradiance level and manufacturers’ spectral irradiance specifications^16–21^ of PT devices was observed. Some PT devices did not meet the AAP-recommended high intensity irradiance limits of ≥30 μW cm^−2^ nm^−1^ ^4,5^. These results may, at least in part, be explained, because manufacturers’ spectroradiometers are non-indentical to the one we used, and different spectroradiometers may yield different irradiance readings for similar PT devices.^33^ In addition, the incubator attenuates the PT light that reaches the newborn with ~4–5% due to Fresnel light reflection on the surface of the plastic incubator.^34^ This attenuation may also explain a small part of the difference in irradiance readings from those reported by the manufacturers, at least when these latter measurements are done without an incubator.

Ideally, PT devices provide a uniform spectral irradiance over the average BSA of a (pre)term infant, but our findings demonstrate that the spectral irradiance is not uniformly distributed. Several studies described, but did not quantify spectral irradiance uniformity.^3,5,7,23^ The method we used can aid to quantify this effect. We measured an intensity decay towards periphery for all PT devices, but only for one device the spectral irradiance uniformity was >10 % of the spectral irradiance.

Although spectral irradiance footprint is essential for PT to be effective^8,25,27^ it is, in analogy to irradiance level, not routinely evaluated in current clinical practice. We measured a large variation in HSI footprints between PT devices and only one PT device covered the average BSA of infants born at 40 weeks of gestation. Therefore, we recommend to perform these footprint measurements in addition to spectral irradiance measurements. Patient-specific PT can be applied by adjusting the distance between the PT device and the patient. By increasing this distance, the spectral irradiance footprint increases, but the spectral irradiance level and HSI footprint decrease, and vice versa.

Several studies reported side effects of previously common fluorescent tube PT devices, including a tendency towards increased mortality in (pre)term newborn infants.^35^ Increased oxidative stress is one of the suggested harmful side effects using fluorescent tube PT devices. A recent study showed that LED-based PT at irradiances up to 35 μW cm^−2^ nm^−1^ given to preterm infants ≤32 weeks of gestation did not affect an oxidative marker of DNA damage.^36^ Currently, no limits are set for the maximum spectral irradiance.^4^ If such limits will be recommended, our proposed method can be used to test whether PT devices comply.

Healthcare providers should be aware of spectral irradiance levels and footprint differences between LED-based PT devices. Therefore, we recommend to perform spectral irradiance measurements in order to enable patient-specific PT, considering PT as a “drug” to be dosed cautiously and appropriately, in analogy to Lamola’s pharmacologic view of PT.^6,9,10^ We also recommend to include in hyperbilirubinemia guidelines not only to perform spectral irradiance measurements^14,15,37^ but also a standard method to perform these and HSI footprint measurements.

### Study limitations

To define the number and locations of spatial irradiance measurements and HSI footprint, we had to make a trade-off between spatial resolution (or number of measurements) and user friendliness. We used five measurement locations to define spectral irradiance, because this covers a large part of the average BSA of a term newborn infant. Furthermore, we used four measurement locations to define HSI footprint because irradiance decreases from the center to the periphery of the device.^4,5^ We think that increasing the number of measurement points between maximum spectral irradiance >30 μW cm^−2^ nm^−1^ (center) and 30 μW cm^−2^ nm^−1^ (periphery) will not add crucial information to adjust intensive PT.

The measured irradiance footprint is not identical to BSA illumination, because the newborn’s skin is not uniformly illuminated by a PT device. Irradiance footprints from overhead devices may underestimate the irradiance of areas which are illuminated closer to the PT device than the mattress. Conversely, irradiance footprints may overestimate irradiance received by skin areas at the sides of the body. Oblique illumination with respect to the skin surface can induce shadow formation or reduce the penetration depth of the PT light into the skin. Thus, irradiance footprints may overestimate irradiance received by skin areas at the sides of the body. To adjust for lower irradiance towards the curved edges of the body, Hart et al.^7^ used a correction factor between 0.5 and 0.8 for these areas in the calculation of effective irradiance. In addition, the BSA silhouette model has some limitations as the arms and legs of the newborn infant are extended. Flexion of the extremities is also a common position, may increase BSA in HSI footprint.

As we included one PT device per brand, we were not able to evaluate the inter-brand reproducibility. This inter-brand variability of PT devices is an interesting direction of future studies.

### Clinical implications

This study provides insight into the spectral irradiance level and HSI footprint of six different brands of PT devices. Our findings demonstrate that some commercially available PT devices have lower spectral irradiance levels than the manufacturer’s specifications, and spectral irradiance levels does not always meet the recommended high spectral irradiance of ≥30 μW cm^−2^ nm^−1^. Furthermore, HSI footprints do not always cover the entire average BSA of a newborn infant.

The scope of this study was to evaluate the technical factors of the PT device itself. In clinical practice, the effectiveness of the phototherapy depends not only on PT device performance but also on how well the phototherapy photons can be delivered to the bilirubin molecules inside the skin of the patient.^9^ This is influenced, among other factors such as the bilirubin level, by the optical properties of the skin, and the percentage of exposed skin (free from diapers, bandages, etcetera). We recommend to perform spectral irradiance and HSI footprint measurements of PT devices on a regular base, i.e., before PT is started and preferably also during PT.

## Conclusion

In this study, we report a clinically relevant and user-friendly method to reliably evaluate spectral irradiance level and HSI footprint of PT devices. We recommend this measurement method to customize PT covering the entire average BSA of a pre(term) newborn infant with sufficient irradiance. Implementation of this method will contribute to awareness of the importance of irradiance level and footprint measurements in the management of neonatal jaundice.

## References

[1] Le Pichon JB, Riordan SM, Watchko J, Shapiro SM (2017). The neurological sequelae of neonatal hyperbilirubinemia: definitions, diagnosis and treatment of the kernicterus spectrum disorders (KSDs). Curr. Pediatr. Rev..

[2] Maisels MJ, McDonagh AF (2008). Phototherapy for neonatal jaundice. N. Engl. J. Med..

[3] Bhutani VK (2011). Phototherapy to prevent severe neonatal hyperbilirubinemia in the newborn infant 35 or more weeks of gestation. Pediatrics (Evanst.).

[4] American Academy of Pediatrics Subcommittee on Hyperbilirubinemia. (2004). Management of hyperbilirubinemia in the newborn infant 35 or more weeks of gestation. Pediatrics.

[5] Vreman HJ, Wong RJ, Stevenson DK (2004). Phototherapy: current methods and future directions. Semin. Perinatol..

[6] Ebbesen F, Hansen TWR, Maisels MJ (2017). Update on phototherapy in jaundiced neonates. Curr. Pediatr. Rev..

[7] Hart G, Cameron R (2005). The importance of irradiance and area in neonatal phototherapy. Arch. Dis. Child Fetal Neonatal Ed..

[8] Dicken P, Grant LJ, Jones S (2000). An evaluation of the characteristics and performance of neonatal phototherapy equipment. Physiol. Meas..

[9] Lamola AA (2016). A pharmacologic view of phototherapy. Clin. Perinatol..

[10] Hansen TWR (2020). Sixty years of phototherapy for neonatal jaundice—from serendipitous observation to standardized treatment and rescue for millions. J. Perinatol..

[11] van Erk MD (2019). How skin anatomy influences transcutaneous bilirubin determinations: an in vitro evaluation. Pediatr. Res.

[12] Bosschaart N, Mentink R, Kok JH, van Leeuwen TG, Aalders MC (2011). Optical properties of neonatal skin measured in vivo as a function of age and skin pigmentation. J. Biomed. Opt..

[13] Bhutani VK (2013). Predischarge screening for severe neonatal hyperbilirubinemia identifies infants who need phototherapy. J. Pediatr..

[14] van Imhoff DE (2013). High variability and low irradiance of phototherapy devices in dutch NICUs. Arch. Dis. Child. Fetal Neonatal Ed..

[15] Hulzebos CV, van’t Klooster SJ, Lorenz K, Vreman HJ, Dijk PH (2017). Irradiance levels of phototherapy devices: a national study in dutch neonatal intensive care units. J. Perinatol..

[16] General Electric Company. *Giraffe™ blue spot PT lite™ Operation, Maintenance, and Service Manual* (2020).

[17] Arseus Medical. *Infantulus, Super-LED Phototherapy* (2015).

[18] Draeger Medical Systems I. *Instructions for Use BiliLux, LED Phototherapy Light, Software 1.n* (2017).

[19] Natus Medical Incorporated. *neoBLUE® LED Phototherapy System User Manual* (2015).

[20] General Electric Company. *Lullaby™ LED Phototherapy System Service Manual* (2019).

[21] Löwenstein Medical. *Bilibluelight Phototherapy System, Reliable Phototherapy for Our Smallest Patients* (2018).

[22] Sampurna MTA (2019). Current phototherapy practice on Java, Indonesia. BMC Pediatr..

[23] Vreman HJ, Wong RJ, Murdock JR, Stevenson DK (2008). Standardized bench method for evaluating the efficacy of phototherapy devices. Acta Paediatr..

[24] Vandborg PK, Hansen BM, Greisen G, Ebbesen F (2012). Dose-response relationship of phototherapy for hyperbilirubinemia. Pediatrics.

[25] Tan KL (1991). Phototherapy for neonatal jaundice. Clin. Perinatol..

[26] Mims LC, Estrada M, Gooden DS, Caldwell RR, Kotas RV (1973). Phototherapy for neonatal hyperbilirubinemia–a dose: response relationship. J. Pediatr..

[27] Maisels MJ (2001). Phototherapy–traditional and nontraditional. J. Perinatol..

[28] Reda SM, Faramawy SM (2020). Developing an automated transition stage as a way to improve the quality of the measurements accuracy. IEEE Instrum. Meas. Mag..

[29] General Electric. *BiliBlanket Meter II Specifications* (2011).

[30] University of Oxford. *Intergrowth Very Preterm*; http://intergrowth21.ndog.ox.ac.uk/Content/PDF/VeryPreterm/INTERGROWTH-21st_Length_Standards_Boys.pdf. (assessed at 3-6-2021).

[31] University of Oxford. *Intergrowth NewBorn*. http://intergrowth21.ndog.ox.ac.uk/Content/PDF/NewBorn/INTERGROWTH-21st_Length_Standards_Boys.pdf. (assessed at 3-6-2021).

[32] Riddle, W. R. & DonLevy, S. C. Continuously tracking growth of preterm infants from birth to two years of age. *J. Neonatol. Clin. Pediatr*. 10.24966/NCP-878X/100011 (2015).

[33] Reda SM, AbdElmageed AA, Monem AS, El-Gebaly RH, Faramawy SM (2018). Estimation of spectral mismatch correction factor f1’ indicated by radiometer responsivity toward phototherapic infant devices. Appl. Opt..

[34] Honsberg, C. B. & Bowden S. G. *Fresnel Reflection calculator (n plastic=1.5), Photovoltaics Education Website* (updated 2019); www.pveducation.org.

[35] Morris BH (2008). Aggressive vs. conservative phototherapy for infants with extremely low birth weight. N. Engl. J. Med.

[36] van der Schoor LWE (2020). Blue LED phototherapy in preterm infants: effects on an oxidative marker of DNA damage. Arch. Dis. Child Fetal Neonatal Ed..

[37] Sampurna MTA (2020). An evaluation of phototherapy device performance in a tertiary health facility. Heliyon.

